# Breathlessness assessment, management and impact in the intensive care unit: a rapid review and narrative synthesis

**DOI:** 10.1186/s13613-024-01338-7

**Published:** 2024-07-05

**Authors:** Ben R. Richardson, Maxens Decavèle, Alexandre Demoule, Fliss E. M. Murtagh, Miriam J. Johnson

**Affiliations:** 1 School of Health and Life Sciences, Teesside University, Tees Valley, Middlesbrough, TS1 3BX UK; 2 Sorbonne Université, INSERM, UMRS1158 Neurophysiologie Respiratoire Expérimentale et Clinique, 75005 Paris, France; 3 Groupe Hospitalier Universitaire APHP-Sorbonne Université, Site Pitié-Salpêtrière, Service de Médecine Intensive et Réanimation (Département R3S), 75013 Paris, France; 4 Wolfson Palliative Care Research Centre, Hull York Medical School, University of Hull, Cottingham Road, Hull, HU6 7RX UK

**Keywords:** Intensive care unit, Breathlessness, Dyspnoea, Invasive mechanical ventilation, Non-invasive ventilation, Rehabilitation, End-of-life

## Abstract

**Background:**

Adults in the intensive care unit (ICU) commonly experience distressing symptoms and other concerns such as pain, delirium, and breathlessness. Breathlessness management is not supported by any ICU guidelines, unlike other symptoms.

**Aim:**

To review the literature relating to (i) prevalence, intensity, assessment, and management of breathlessness in critically ill adults in the ICU receiving invasive and non-invasive mechanical ventilation (NIV) and high-flow oxygen therapy, (HFOT), (ii) the impact of breathlessness on ICU patients with regard to engagement with rehabilitation.

**Methods:**

A rapid review and narrative synthesis using the Cochrane Methods Group Recommendations was conducted and reported in accordance with PRISMA. All study designs investigating breathlessness in adult ICU patients receiving either invasive mechanical ventilation (IMV), NIV or HFOT were eligible. PubMed, MEDLINE, The Cochrane Library and CINAHL databased were searched from June 2013 to June 2023. Studies were quality appraised.

**Results:**

19 studies representing 2822 ICU patients were included (participants mean age 48 years to 71 years; proportion of males 43–100%). The weighted mean prevalence of breathlessness in ICU patients receiving IMV was 49% (range 34–66%). The proportion of patients receiving NIV self-reporting moderate to severe dyspnoea was 55% prior to initiation. Breathlessness assessment tools included visual analogue scale, (VAS), numerical rating scale, (NRS) and modified BORG scale, (mBORG). In patients receiving NIV the highest reported median (interquartile range [IQR]) VAS, NRS and mBORG scores were 6.2cm (0–10 cm), 5 (2–7) and 6 (2.3–7) respectively (moderate to severe breathlessness). In patients receiving either NIV or HFOT the highest reported median (IQR) VAS, NRS and mBORG scores were 3 cm (0–6 cm), 8 (5–10) and 4 (3–5) respectively.

**Conclusion:**

Breathlessness in adults receiving IMV, NIV or HFOT in the ICU is prevalent and clinically important with median intensity ratings indicating the presence of moderate to severe symptoms.

**Supplementary Information:**

The online version contains supplementary material available at 10.1186/s13613-024-01338-7.

## Introduction

Adults admitted to the intensive care unit (ICU) commonly experience distressing symptoms and other concerns such as pain, thirst, anxiety, agitation, sleep disturbance, delirium and immobility and breathlessness, causing suffering and potential barriers to rehabilitation during the ICU stay [1, 2]. Also, for those admitted to the ICU who die in the unit, symptom identification and control is crucial [3].

Besides generating immediate and intense fear and distress in ICU patients receiving mechanical ventilation, breathlessness (defined as a subjective experience of breathing discomfort [4], and medically known as dyspnoea) is associated with serious unfavourable consequences, such as an associated higher-risk of weaning failure during a spontaneous breathing trial, (SBT) [5] and post-traumatic stress disorders [6]. Yet, unlike other symptoms such as pain [7], and despite an evidence-base for breathlessness management in general [8], in the ICU setting [9] breathlessness management is not supported by any guidelines [10].

Breathlessness might also delay or prevent rehabilitation in the ICU. From the pulmonary rehabilitation (PR) literature, we know that people with moderate to severe breathlessness are less likely to attend or complete PR [11, 12], and there is a recognised vicious cycle of breathlessness, avoidance of physical exertion, worsening deconditioning, and worsening breathlessness [8, 13, 14]. Known barriers to rehabilitation within the ICU [2, 15] include respiratory instability [2, 16], insufficient respiratory reserve [17], respiratory distress [2] and ventilator asynchrony [2]. However, to our knowledge, little is published about the impact of patient self-reported breathlessness levels on patient participation in rehabilitation or levels of physical function during the ICU stay.

This lack of attention raises concerns that adult ICU patients have sub-optimally managed breathlessness, causing (i) suffering for all (patients and family caregivers), including those who are dying, and (ii) a barrier for rehabilitation, particularly for those already at high risk due to pre-existing conditions and frailty [18, 19].

A recent systematic review and narrative synthesis summarises the literature on the prevalence, intensity, assessment, consequences, and management of breathlessness in acutely ill invasively mechanically ventilated adults [20]. Building on this work, we aimed to conduct a rapid review and narrative synthesis of the literature relating to (i) prevalence, intensity, current identification and assessment of breathlessness, and management of breathlessness in critically ill adults in the ICU receiving invasive and non-invasive mechanical ventilation (ii) the impact of breathlessness on ICU patients with regard to engagement with rehabilitation.

## Methods

This unregistered narrative rapid review was guided by the Cochrane Rapid Reviews Methods Group Recommendations [21] and reported in accordance with the Preferred Reporting Items for Systematic Reviews and Meta-Analyses 2020, (PRISMA) statement [22].

### Eligibility criteria

Articles retrieved through the literature search were potentially eligible for inclusion if they met the criteria listed in the Table 1.

**Table 1:** Inclusion and exclusion criteria

Inclusion criteria	Exclusion criteria
Population: adult inpatients (18 + years), in the intensive care unit during their admission Exposure: receiving High Flow Oxygen Therapy (HFOT), Non Invasive Ventilation (NIV) or Invasive Mechanical Ventilation (IMV) for acute respiratory failure Outcome: breathlessness presence / absence and intensity Study Types: all empirical study designs (quantitative—including observational and experimental—and qualitative); articles published after May 2013 to gain/optimize information on current practice	1. Articles not published in a peer reviewed journal 2. Non-English publications 3. Textbooks, opinion pieces and study protocols, case histories 4. Grey literature (materials published outside of academic publishing and distribution channels) 5. COVID-19 related articles 6. Participants aged under 18 years of age

### Information sources

PubMed, MEDLINE, The Cochrane Library and CINAHL databased were searched by BR from 01/06/2013 to 30/06/2023. Bibliographies of included studies were also searched. The systematic review [20] conducted as part of The European Respiratory Society, (ERS) / European Society of Intensive Care Medicine, (ESICM) task force on “Dyspnea in critically ill mechanically ventilated patients” was also used as a source of relevant article of ICU patients receiving invasive mechanical ventilation [20].

### Search strategy

The Medical Subject Headings, (MeSH) thesaurus was used to identify all key words specific to “intensive care unit” and “breathlessness/dyspnea” to help balance the risk of excessive articles being retrieved whilst ensuring adequate sensitivity.

Searches used the pre-developed search terms with “Title” as the chosen search field. Search 1” used the following search terms and Boolean operators “intensive care” OR *“critical care”* OR *“critical illness”* OR *“critically ill”* OR *“critically unwell”*. “Search 2” related to concept 2 and used the following search terms and Boolean operators; “breathless*” OR “dyspn*”. Search 3 combined the results of “Search 1” AND “Search 2” together. Filters were used relating to publication date, English language only, participant age and COVID-19.

### Screening and selection process

A single reviewer, (BR) screened the titles and abstracts of the search findings against the eligibility criteria. Full texts of potentially eligible articles published in the English Language were retrieved and screened in full by BR. BR and MJ discussed reasons for exclusion and reviewed any uncertainties. A third reviewer was available for outstanding disagreements but was not required.

### Data collection and management process

BR conducted the data extraction using a standardised data collection template (Microsoft^®^ Excel). Data were summarised in descriptive tables by BR, and a random sample quality checked for accuracy by MJ. Characteristics of included studies (author, year, country, design and sample size) and participants (age, gender, reason for ICU admission and respiratory support received) were extracted. Estimated prevalence and intensity of breathlessness, type of breathlessness assessment tool, timing and frequency of assessment and management of breathlessness were noted. For qualitative data, the themes identified were recorded with illustrative quotes.

### Quality assessment

No risk of bias assessment tool was used but quality was appraised by BR using Critical Appraisal Skills Programme Checklists [23], JBI Critical Appraisal Tools [24] and the results agreed with MJ [23, 24]. Details of the quality appraisal process can be seen in the online supplement. Studies were not excluded on this basis, but the quality was considered in interpreting findings [25].

### Synthesis methods

Included study characteristics are presented descriptively in Table 1 consistent with rapid review methods, meta-analysis of prevalence figures or other quantitative findings was not conducted, but findings are presented as a narrative summary [21]. As only one paper with qualitative data was found, qualitative synthesis was not possible. Prevalence estimates in each paper were weighted according to individual study’s proportion of total sample size and then averaged across all studies.

## Results

### Study selection

**Fig. 1:**
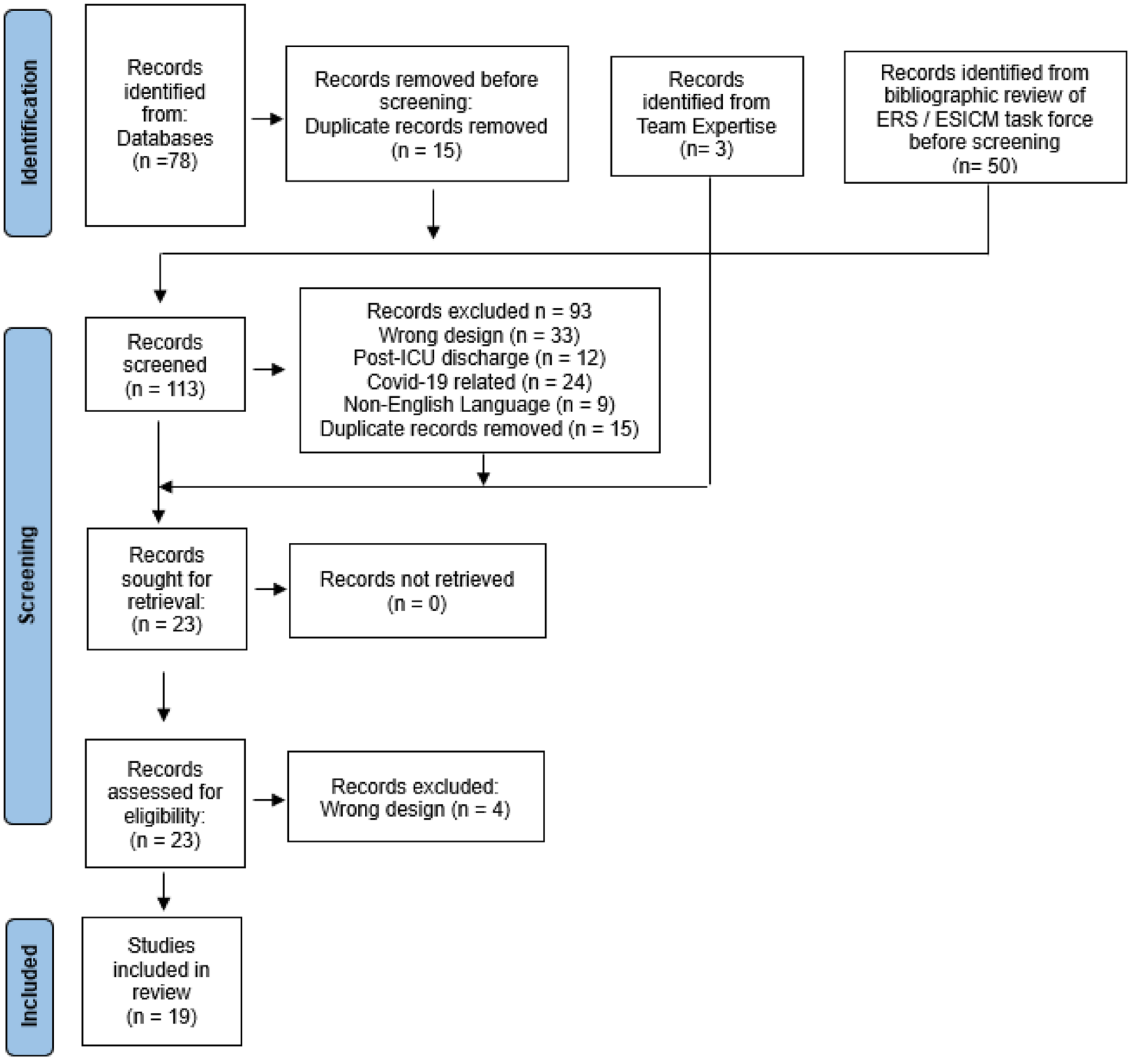
PRISMA flowchart

The PRISMA flowchart diagram is shown in Fig. 1; 113 potential studies were identified of which 78 remained following de-duplication for screening. The full text publications of 21 studies were retrieved and assessed for eligibility; a further 2 studies were excluded on the basis of their design. The reference lists of each of these studies were also reviewed. The final total number of studies included in this rapid review was 19 [6, 26–43].

### Study characteristics

The 19 studies in this rapid review includes 18 quantitative studies [6, 26–42] and 1 mixed methods study [43] (see Table 2). Thirteen quantitative studies used observational methods, including multi-centre observational cohort study (n = 1) [39], multi-centre prospective cohort studies (n = 3) [6, 31, 34], single-centre cohort observational study (n = 2 [30, 33], multi-centre cross-sectional observational Study(n = 1) [38], single-centre cross sectional observational study (n = 1) [27] and single-centre cross sectional observational study (n = 5) [26, 28, 32, 36, 40]. The remaining five quantitative studies used an interventional design; a single-centre randomised controlled trial (RCT) (n = 2) [29, 35], multi-centre RCT (n = 1) [37] and single-centre crossover RCT (n = 2) [41, 42] each testing a different intervention.

**Table 2:** Characteristics of included studies

The quantitative studies						
Characteristics of included studies		Participant characteristics			Intensive care admission history	
Author Year Country	Study design & participants, n	Age mean years (range)	Males, n (%)	Type of ICU patients	ICU admission reason, n (%):	Ventilatory support, n (%):
Bureau 2022 [26] France	Single-centre, Cross Sectional Observational Study 1 ICU n = 31	65 (61–71)	22 (71)	General, Medical	ARF, 22 (71) Coma, 4 (13) Cardiac Arrest, 2 (6) Shock, 2 (6)	Invasive Ventilation, 31 (100%) Modes of Invasive Ventilation: Not specified Pressure Support 7cmH_2_O End Expiratory Pressure 0cmH_2_O
Demoule 2022 [6] France	Multi-centre, Prospective Cohort Study 10 ICU’s n = 612	64 (54–72)	381 (62)	General, Medical & Surgical	A-C-F ARF, 86 (14) Acute CPO, 60 (10) Coma, 58 (10) De novo ARF, 211 (35) Sepsis, septic shock, 77 (13) Postoperative, 30 (5) Other- not specified, 89 (14) ARDS, 217 (36)	Invasive Ventilation, 612 (100) Modes of Invasive Ventilation: AC, 53 (9) PSV, 552 (90) Other mode- not specified, 7 (2)
Sato 2022 [27] Japan	Single-centre, Cross sectional Study 1 ICU n = 184	48 (36–55)	97 (53)	Specialist, Post-operative lung transplant surgery	Reasons for Transplant surgery: ILD, 96 (52) Pulmonary Complications post-HSCT, 29 (16) IPAH, 15 (8) COPD, 13 (7) Lymphangioleiomyomatosis, 11 (6) Bronchiectasis, 11 (6) CF, 2 (1%) Other- not specified, 7 (4)	Invasive ventilation, 10 (5) Modes of Invasive Ventilation- not specified Pre-operative tracheostomy, 8 (4)
Bureau 2021 [28] France	Single-centre, Cross Sectional Observational Study 1 Medical ICU n = 34	66 (57–77)	25 (73)	General, Medical	Bacterial pneumonia, 11 (33) Viral or fungal pneumonia, 4 (12) Aspiration pneumonia, 3 (9) A-C-F RF, 10 (30) Cardiogenic pulmonary oedema, 2 (6) Other- not specified, 4 (12)	Invasive ventilation, 34 (100) Modes of Invasive Ventilation: PSV-B, 31 (100) PSV-P, 31 (100) PAV + , 31 (100)
Yilmaz 2021 [29] Turkey	Single-centre, Randomised Controlled Clinical Trial 1 ICU Intervention Group, n = 49 Control Group, n = 42	70.6 (Not Specified)	36 (62.1)	General, Medical	Acute exacerbation COPD, 58 (100)	NIMV, 58 (100)- intermittent use Modes of Ventilation- not specified Oxygen therapy usage Intervention group, 21 (75); Control group, 21 (70)
Atrous 2020 [30] Egypt	Single-centre, Cohort Observational Study 1 ICU n = 40	53 (Not Specified)	31 (78%)	General	Pulmonary disorders (not specified), 20 (50%)	Invasive Ventilation, 40 (100%) Modes of Ventilation: ACV (not specified) PSV (not specified) BIPAP (not specified) SIMV (not specified) CPAP (not specified)
Mazeraud 2020 [31] France	Multi-centre, Prospective Cohort Study 3 ICU’s n = 391	63 (49–74)	232 (59%)	General, Medical & Surgical	ARF, 127 (33) Sepsis, 74 (19) Surgery, 37 (9) Acute renal failure, 20 (5) Haemodynamic failure, 13 (3) Other- not-specified, 120 (31)	Invasive ventilation, 0 (0) Modes of Ventilation used NIV or HFNOT, 85 (22)
Gentzler 2019 [32] USA	Single-centre, Cross Sectional Observational Study (Secondary data analysis derived from a cohort study) 1 Medical ICU n = 138	64.51 18.44 SD	85 (62)	General, Medical	Respiratory failure, 87 (63) Sepsis / Septic Shock, 19 (14) Hypotension, 21 (15) Cardiac arrest, 7 (5) Other- not specified, 28 (20)	Invasive ventilation, 89 (65) Respiratory device: None, 10 (7) Ventilator- not specified, 89 (65) Other- not specified, 39 (28)
Raux 2019 [33] France	Single-centre, Cohort Observational Study 2 ICU’s n = 12	62 (37–87)	9 (75)	General, Medical & Surgical	Acute kidney injury, 1 (8%) Acute or chronic RF, 2 (17%) Acute pancreatitis, 2 (17%) De novo acute RF, 4 (33%) Peritonitis, 2 (17%) Sepsis, 1 (8%)	Invasive ventilation, 12 (100) Modes of Invasive Ventilation: PSV, 12 (100)
Dangers 2018 [34] France Belgium	Multi-centre, Prospective Cohort study 54 ICU’s n = 426	69 (60–78)	270 (63)	General, Medical	A-C-F, 251 (59) Acute CPO, 58 (14) De novo ARF, 116 (27)	NIV, 426 (100) Mode of Ventilation used: PSV, AC & CPAP
Akoumianaki 2017 [35] Switzerland	Single-centre, Crossover randomised controlled trial 1 ICU n = 10 Intervention Group: PAV + , n = 6 NAVA, n = 4 Control Group: PSV, n = 10	Intervention Group: 58 (53–69) Control Group: 60 (48–72)	7 (70%)	General, Medical & Surgical Difficult to wean	Sepsis, 2 (20) Cardiac Arrest, 1 (10) CO Intoxication, 1 (10) Pneumonia, 2 (20) Oesophageal Cancer, 1 (10) Poly Trauma, 1 (10) ARF, 1 (10) AECOPD, 1 (10)	Invasive Ventilation, 10 (100%) Modes of Invasive Ventilation: PAV +, 6 (60) NAVA, 4 (40)
Binks 2017 [36] USA	Single-centre, Cross Sectional Observational Study Multidisciplinary special care unit n = 30	Data Not Available	22 (73)	General, Medical & Surgical	Airway protection, 2 (7) CHF, 2 (7) COPD, 3 (10) Pneumonia, 4 (13) Post-operative, 7 ((23) Trauma, 11 (39) Asthma, 1 (1)	Invasive ventilation, 30 (100) Modes of Invasive Ventilation: CSV, 15 (50) VC-CMV, 11 (37) APRV, 3 (10) VC-IMV, 1 (3)
Demoule 2016 [37] France	Multi-centre, Randomised Controlled Clinical Trial 11 ICU’s n = 128 Intervention Group, n = 62 Control Group, n = 66	Intervention Group: 66 (61–77) Control Group: 64 (53–77)	Intervention Group: 47 (76) Control Group: 39 (59)	General, Medical & Surgical	Intervention Group: De novo RF, 34 (55): Pneumonia, 21 Aspiration, 4 Extra-pulmonary sepsis, 3 Other, 6 Post-operative, 13 (21): Pneumonia, 3 Extra-pulmonary sepsis, 3 ARDS post-cardiac surgery, 4 Haemorrhagic shock / trauma, 2 Other, 1 A-C-F, 12 (19) Acute CPO, 3 (5) Control Group: De novo RF, 38 (58): Pneumonia, 23 Aspiration, 7 Extra-pulmonary sepsis, 3 Other, 5 Post-operative, 13 (20): Pneumonia, 3 Extra-pulmonary sepsis, 2 ARDS post-cardiac surgery, 4 Haemorrhagic shock / trauma, 2 Other, 2 A-C-F, 12 (18) Acute CPO, 3 (5)	Invasive Ventilation, 128 (100%) Modes of Ventilation: Intervention Group- NAVA, 62 (48) Control Group- PSV, 66 (52)
Schmidt 2016 [38] France and Belgium	Multi-centre, Cross-Sectional Observational Study 50 ICU’s n = 396	69 (60–80)	226 (57)	General, Medical & Surgical	Decompensation of Chronic Respiratory Disease, 260 (67) De Novo ARF, 136 (33)	Non-Invasive Ventilation, 396 (100) Mode of Non-Invasive Ventilation used: Not Specified
Haugdahl 2015 [39] Norway	Multi-centre, Observational Cohort Study 3 ICU’s n = 100	65 (58–74)	56 (56%)	General, Medical & Surgical	Respiratory, 37 (37) Cardiovascular, 20 (20) Infection, 18 (18) Gastrointestinal, 16 (16) Trauma, 6 (6) Renal, 2 (2) Other- not specified, 1 (1)	Invasive ventilation n = 100 (100%) pre-SBT Modes of Invasive Ventilation: PSV (PS 6–8 cmH2O) & (PEEP 6–8 cmH_2_O) T-piece circuit External CPAP with PEEP (5 cmH_2_O)
Persichini 2015 [40] France	Single-centre, Cross Sectional Observational Study 1 ICU n = 220	61 (46–71)	72 (60%)	General- not specified	Respiratory admission (not specified), 74 (62)	Invasive ventilation 14 (12) Modes of Invasive Ventilation: Invasive ventilation modes (not specified) Oxygen therapy, 80 (67)
Fortis 2015 [41] USA	Single-centre, Crossover randomised controlled trial 1 ICU n = 20 Group 1 (COPD): n = 13 Group 2 (OHS): n = 7	Group 1: 70 (54–89) Group 2: 65 (36–87)	8 (40)	General, Medical	Group 1 (COPD), 13 (65) Pneumonia, 5 Unknown, 1 Urosepsis, 1 Sepsis, 1 Angioedema, 1 CHF, 4 Group 2 (OHS), 7 (35) Pneumonia, 1 Unknown, 1 Urosepsis, 1 CHF, 3 MI, 2	Invasive Ventilation, 20 (100) Modes of Ventilation: 3 min trials repeated 11 separate times AC (8 different trials) PSV (3 different trials)
Vitacca 2014 [42] Italy	Single-centre, Crossover randomised controlled trial Difficult to wean referral centre n = 8 Group Setting-1: n = 5 Group Setting-2: n = 3	67 6 SD	8 (100)	Specialist, COPD	Diagnosis not specified	Invasive ventilation n = 8 (100%) Tracheostomy n = 8 Modes of Invasive Ventilation: Group Setting-1; 30 min PSV 20 cmH_2_O + PEEP 0 cmH_2_O Group Setting-1; 30 min PSV 15 cmH_2_O + PEEP 5 cmH_2_O

The Survey and qualitative data study				
Characteristics of included studies		Design and methods		Participant characteristics
Author Year Country	Study aim and location	Sampling approach	Data collection and mixed methods analysis	Study participants, (n)
Baker 2020 [43] USA	To assess nurses’ perceptions of the utility of routine dyspnea measurement, patients’ comprehension of assessment questions, and the impact on nursing practice and to gather nurses’ suggestions for improvement Academic Tertiary Care Hospital with 8 separate ICU’s covering all general and specialist ICU.	Convenience sampling approach ICU Nurses working in an academic Tertiary Care Hospital with 8 ICU’s invited to take part in focus groups or anonymous online survey	Focus Groups: Led by 2 research Nurses 2 separate Face to face 30-min focus groups Focus groups recorded and transcribed verbatim Structured interview guide used Only nurses on duty eligible to participate No data analysis methods described Anonymous online survey: Sample size n = 48 14% of total nursing workforce randomly selected from each of the 8 ICU’s Invitations sent via email with reminders for non-completers Descriptive statistical data analysis used	Focus Groups Participants, (n): Focus Group one, (7); Focus Group two, (10)- no further participant information specified Online Survey: 37 (77%) completion rate, of these: 17 (46% had been an ICU Nurse > 10 years 14 (38%) had worked at the hospital > 10 years

*AC* assist-control, *A-C-F* acute-on-chronic, *ACV* assist-control ventilation, *AE* acute exacerbation, *APRV* airway pressure release ventilation, *ARDS* acute respiratory distress syndrome, *ARF* acute respiratory failure, *BIPAP* biphasic positive airway pressure, *CO* carbon monoxide, *CF* cystic fibrosis, *CHF* congestive cardiac failure, *COPD* chronic obstructive pulmonary disease, *CPAP* continuous positive airway pressure, *CPO* cardiogenic pulmonary oedema, *CSV* continuous spontaneous ventilation, *DNMD* decompensation of neuromuscular disease, *HFNOT* high-flow nasal oxygen therapy, *HSCT* haematopoietic stem cell transplantation, *ICU* intensive care unit, *ICU’s* intensive care units, *ILD* interstitial lung disease, *IPAH* idiopathic pulmonary arterial hypertension, *NAVA* neurally adjusted ventilatory assist, *NIMV* non-invasive mechanical ventilation, *NIV* non-invasive ventilation, *PAV +* proportional assist ventilation, *PEEP* positive end expiratory pressure, *PS* pressure support, *PSV* pressure support ventilation, *PSV-B* pressure support ventilation- baseline, *PSV-P* pressure support ventilation- personalisation, *RF* respiratory failure, *SIMV* synchronised intermittent mandatory ventilation, *SBT* spontaneous breathing trial, *VC-CMV* volume control continuous mandatory ventilation, *VC-IMV* volume control intermittent ventilation

One study of ICU nurses [43] used face-to-face focus groups and an anonymous online survey.

### Quality appraisal

The observational nature of the quantitative studies carries the inherent limits regarding evaluating causality. In general, they were well conducted (see Online Supplemental Tables 1–4) but some lacked consecutive samples and had poor accounting for confounders.

The multi-centre randomised controlled trial [37] comparing neurally adjusted ventilatory assist, (NAVA) ventilation to usual care using pressure support ventilation, (PSV) in the early weaning phase in mechanically ventilated adults had a robust design including an adequately powered sample size, was rigorously conducted, controlled for confounding variables, and reported according to CONSORT [44]. The single-centre randomised controlled trials [29, 35, 41, 42] had limitations including a small sample size, lack of clarity in relation to recruitment, selection, randomisation, and usual care provided. The staff delivering the intervention also undertook the outcome assessments risking reporting bias [29] and were not comprehensively reported according to CONSORT [29, 35, 41, 42].

The study using survey and qualitative data collection had no clear description of the design, comparative weighting given to each type of data gathered, or analysis methods including synthesis methods of the qualitative and quantitative data [43]. Thus the quantitative and qualitative components were appraised separately, as it was not clear if this was designed as a mixed-methods study. Focus group participants (ICU nurses) formed a convenience sample (only those working on a particular day). The anonymous online survey had a target sample size which only represented 14% of the total ICU nurse workforce in the hospital, and no rationale was given for the chosen sample size [43].

### Participants

Included studies represented 2822 critically ill adults (age range 36 [27] to 89 years [41]; proportion male 43% [41] to 100% [42]). Sample sizes ranged from n = 8 [42] to n = 612 [6]. The mean average age was reported in 17/19 studies and ranged from 48 years [27] to 71 years [29]. Most studies were set in a General ICU [26, 28–41] with 2/16 in a specialist ICU [27, 42]. Patients were categorised into three groups—medical only, medical and surgical or surgical only. Seventeen of the 19 studies reported data on these sub-categories with most of these being medical [26, 28, 29, 32, 34, 41, 42] or medical-surgical [6, 31, 33, 35–39]. The four most frequently reported reasons for ICU admission were respiratory-related (See Table 2).

All quantitative studies described the level of respiratory support needed during the ICU admission; IMV (15/18) [6, 26–28, 30–37, 39–41]; NIV (3/18) [29, 34, 38]; NIV or HFOT (1/18) [31]. Just over half (10/19) provided detailed information about the modes and settings of ventilatory supported [6, 26, 28, 30, 33, 35–37, 39, 41].

The mixed-methods study [43] recruited n = 17 ICU nurse participants for two focus groups (group 1, n = 7; group 2, n = 10). Demographic participant data was not provided. The anonymous online survey achieved a 77% response rate (n = 37/48 questionnaires), with 17/37 (46%) and 14/37 (38%) having worked as an ICU nurse and or worked at the hospital for ≥ 10 years respectively.

### Prevalence of breathlessness in the ICU

Patient self-reported breathlessness prevalence data was provided by 9/16 studies [6, 27, 30, 32, 33, 37, 39, 40], only one of which related to patients receiving NIV [34] (Table 3).

**Table 3:** Weighted mean average prevalence of breathlessness in patients receiving invasive mechanical ventilation

Reference	Total patients reporting breathlessness, n = (%)	Adjusted weighting	Adjusted breathlessness prevalence, (%)
6	208 (34)	0.40	13.47%
27	116 (63)	0.22	13.92%
30	22 (55)	0.04	2.30%
32	24 (47)	0.05	2.15%
33	5 (37)	0.01	0.35%
37	19 (66)	0.04	2.39%
39	62 (62)	0.12	7.32%
40	69 (57)	0.13	7.49%
Total Patients, (n =):	525	Weighted Mean Average, (%):	49.40

The weighted mean prevalence of breathlessness for patients receiving IMV was 49% (range 34% [6] to 66% [37]). The proportion of patients receiving NIV self-reporting moderate to severe dyspnoea was 55% prior to initiation reducing to 39% after their first NIV session [34].

One study compared patient, caregiver and nurse breathlessness assessments (present/absent) [32]. The prevalence rates of moderate to severe breathlessness was 47% (patients), 61% (caregivers; Cohen’s k coefficient 0.65 (95% confidence interval [CI], 0.40–0.90; p = 0.001), and 34% (nurses: Cohen’s k coefficient 0.19 (95% CI, 20.10–0.48; p = 0.39).

### Assessment of breathlessness in the ICU

Details of the breathlessness assessment approaches in all the 18 quantitative studies [6, 26–42] are presented in Table 4. The choice of assessment tool, timing, and frequency of assessment and the rater varied significantly.

**Table 4:** The prevalence, assessment and management of breathlessness in the ICU: quantitative studies

Author year Country	Assessment tool used	Timing and frequency of assessment	Prevalence, n (%)	Intensity ratings	Management
Bureau 2022 [25] France	VAS: 0–10 cm VAS scores ≥ 4 clinically significant breathlessness	Twice Patient SBT Start & Finish	Not specified	Initiation SBT VAS scores: All patients (n = 31): Median 2 (IQR 0–2) Successful Patients (n = 17): Median 0 (IQR 0–2) Unsuccessful Patients (n = 14): Median 2 (IQR 2–5); p = 0.188 End of SBT VAS Scores All patients (n = 31): Median 4 (IQR 0–10) Successful Patients (n = 17): Median 0 (IQR 0–4) Unsuccessful Patients (n = 14): Median 10 (IQR 8–10); p = 0.003 Change in VAS Scores between SBT Initiation and End: Successful SBT: Median 0 (IQR 0–1) Unsuccessful SBT: Median 6 (4–8); p = 0.006	Not specified
Demoule 2022 [5] France	VAS: 0–10 cm scale VAS scores ≥ 4 clinically significant breathlessness	Once, pre-SBT Patient Daily Assessments up to extubation	208 (34)	Median rating 5 (IQR 4–7) 75% of patients with dyspnoea rated ≥ 4	Not specified
Sato 2022 [26] Japan	Terms searched for in nursing documentation: Dyspnoea; Shortness of breath; Breathlessness & Patient comments	Not-specified	116 (63)	Median frequency of dyspnoea 3 (IQR 1.5)	Not specified
Bureau 2021 [27] France	VAS: 0–10 cm scale IC-RDOS; observational scale; Based on 5 physical and observational signs VAS scores ≥ 4 clinically significant breathlessness IC-RDOS score of ≥ 2.4 predicts a VAS score at least ≥ 4	Once, SBT Start & Finish Patient	Not specified	VAS Scores: PSV-P 37 [IQR 20‒55], p = 0.001) PAV + 31 [IQR 14–45], p = 0.001) PSV-B 62 [IQR 28‒76], p = 0.001) No significant difference between PSV-P & PAV + IC-RDOS Scores: PAV-P 4.18 [IQR 2.36‒4.71], p = 0.002 PSV-B 4.76 [IQR 4.11‒6.46], p = 0.002 PSV-P 4.35 [IQR 2.39‒4.92] IC-RDOS scores were lower with PAV + vs PSV- B, but no significant difference to PSV-P	Ventilator Management: Changing mode & settings Optimising ventilator settings
Yilmaz 2021 [28] Turkey	BDI: 0–12 with 3 different components Functional impairment; Magnitude of effort & Magnitude of task Lower BDI scores indicate greater intensity of breathlessness	Once Daily Patient Pre & Post-intervention Over 4 days	Not specified	Intervention group: Pre-test BDI Scores; 3 (IQR 0.0: 3.0–3.0) Post-test BDI Scores; 3 (IQR 0.0: 3.0–3.0) Tests within group W* = 0.486, p = 0.627 Control Group: Pre-Test BDI Scores; 3 [IQR 0.3: 3–3.3] Post-Test BDI Scores; 3 [IQR 0.0:3–3] Tests within group W* = 0.345, p = 0.730 Between Group Comparison: Pe-test BDI Scores; U = 378.0 p = 0.459 Post-test BDI Scores; U = 394.5 p = 0.639	NIMV stopped during massage Patient re-positioning Back Massage
Atrous 2020 [29] Egypt	mBORG: 0 to 10 mBORG scores < 4 defined as “Mild or No breathlessness” mBORG scores ≥ 4 defined as “Moderate to Severe breathlessness”	Patient Once	55%	Mean mBORG rating was 6.0 (± 1.8)	Not specified
Mazeraud 2020 [30] France	VAS: 0–10 cm scale VAS scores ≥ 4 clinically significant breathlessness	Once daily Patient Assessments over 7 days	Not specified	Median VAS rating was 3 (IQR 0–6) Patients with a STAI < 40, (n = 188) Median D-VAS rating 2 (IQR 0–5) Patients with a STAI > 40, (n = 203) D-VAS Median rating 5 (IQR 1–8) p ≤ 0.0001	Not specified
Gentzler 2019 [31] USA	NRS: 0–10 scale NRS scores ≥ 4 moderate to severe breathlessness	Once: Patient Caregiver Nurse	24 (47) of patients self-reported NRS scores ≥ 4- moderate to severe dyspnoea	Caregiver: 62 (61%) caregiver reported breath-lessness present (Cohen’s k coefficient 0.65 (95% confidence interval [CI], 0.40–0.90; p = 0.001), Nurses: 46 (34%) nurse reported breath-lessness present (Cohen’s k coefficient 0.19 (95% CI, 20.10 to 0.48; p = 0.39)	Pharmacological: Opioids, Benzodiazepines or Inhaled bronchodilators Oxygen delivery devices: Increased in oxygen delivery Change of delivery device Ventilator management: Change of mode, ventilator pressure settings and volumes
Raux 2019 [32] France	VAS: 0–10 cm scale MV-RDOS: observational scale 5 item tool on physical & observational signs VAS scores ≥ 4 clinically significant dyspnoea MV-RDOS ≥ 2.6 predicts a dyspnoea-VAS ≥ 4 with 94% specificity and 57% sensitivity	Once: Patient	Baseline data: 2 (17) patients self-reported breathlessness VAS scores ≥ 4 3 (25) MV-RDOS scores ≥ 2.6	Communicative Patients: Median VAS rating was 3 (IQR 2.5–4) Non-Communicative Patients: MV-RDOS scores 2.9, 2.9 & 2.7	Ventilator management: Change of mode, ventilator pressure settings and volumes
Dangers 2018 [33] France & Belgium	mBORG: 0 to 10 mBORG scores < 4 defined as “Mild or No breathlessness” mBORG scores ≥ 4 defined as “Moderate to Severe breathlessness”	Three Times: Patient ICU Admission Pre-initiation of NIV After first NIV session	234 (55) self-reported breathlessness prior to NIV inhiation 166 (39) self-reported breathlessness after first NIV session	Prior to NIV initiation: mBORG median score 4 (IQR 3–5) After the first NIV session: mBORG median score 3 (IQR 2–4) p < 0.001 NIV failure in patients with moderate-to-severe dyspnoea after the first NIV session (OR 2.41 (95% CI 1.49–3.91), p < 0.0001)	Not specified
Akoumianaki (2017) [34] Switzerland	mBORG: 0 to 10 mBORG scores < 4 defined as “Mild or No breathlessness” mBORG scores ≥ 4 defined as “Moderate to Severe breathlessness”	Three times: Patient Baseline exercise test PAV + session NAVA session	Not specified	Baseline: mBORG median score 4.5 (IQR 2.3–5.0) PSV ventilation mBORG median score 3.5 (IQR 1.3–6) NAVA / PAV + ventilation During Exercise: mBORG median score 5 (IQR 4.3–6) PSV ventilation mBORG median score 3.5 (IQR 1.3–6) NAVA / PAV + ventilation Ventilator mode had no statistically significant effect on dyspnea; PSV median mBORG scores 2 (IQR 0.5–4) and PAV + / NAVA median mBORG score 1 (IQR 0.8–3.8) p = 0.33	Not specified
Binks 2017 [35] USA	mBORG; 0 to 10 mBORG scores ≥ 4 or”somewhat severe” indicate clinically significant breathlessness	Once: Patient Nurse / Physician / Respiratory Therapist	Not specified	mBORG median score was 4 (IQR 4–7) Clinicians underestimated patient breathing discomfort by a median (IQR) of 2 scale points (0 –3)	Not specified
Demoule 2016 [36] France	VAS: 0–10 cm scale VAS scores ≥ 4 clinically significant breathlessness	Three times Patient Day 1 Day 2 Day 5	Intervention Group: Day 1, 9 (28) Day 2, 14 (50) Day 5, 3 (20) Control Group: Day 1, 19 (66) Day 2, 13 (52) Day 5, 6 (46) p = 0.67	Not specified	Not specified
Schmidt 2016 [37] France and Belgium	NRS: 0–10 scale NRS scores ≥ 4 moderate to severe breathlessness	Patient Relatives Once- Retrospectively	Not specified	Patients NRS Scores: Felt dyspnoeic during NIV No / low level of anxiety during NIV; Median 3 (IQR 1–7) High level of anxiety during NIV; Median 8 (IQR 5–10); p ≤ 0.0001 (OR 1.16 (95% CI 1.06–1.26), p < 0.0010) Relatives: Felt that the patient was dyspnoeic during NIV No / low level of anxiety; Median 2 (IQR 1–5) High level of anxiety during NIV; Median 5 (IQR 2–8), p ≤ 0.002	Not specified
Haugdahl 2015 [38] Norway	NRS: 0–10 scale NRS scores ≥ 4 moderate to severe breathlessness	End of SBT Patient Nurse Physician	62 (62) patients self-reported moderate to severe breathlessness	The median patient self-reported breathlessness rating 5 (IQR 2–7) Median Nurse rated breathlessness scores was 2 (IQR 0–3.75) & Physicians 2 (1–4) p = 0.001 54% of Nurses & 48% of Physicians underestimated breathlessness compared with the patients self-reports	Not specified
Persichini 2015 [39] France	VAS: 0–10 cm scale VAS scores ≥ 4 clinically significant dyspnoea	Once Patient	69 (57) patients self-reported moderate to severe breathlessness	The median patient self-reported breathlessness rating was 4.5 (IQR 3.2–6.0)	Not specified
Fortis [40] 2015 USA	mBORG; 0 to 10 mBORG scores ≥ 4 or “somewhat severe” indicate clinically significant dyspnoea	Multiple patient	Not specified	No specific ventilator settings proved superior AC: Minimum range mBORG scores 0 to 1 Maximum range mBORG scores 0 to 8 PSV: Minimum and Maximum range mBORG “similar results noted” not specified	Ventilator management: Change of ventilator pressure settings, flows and volumes
Vitacca 2014 [41] Italy	VAS: 0–20 cm scale Reference range not specified	Multiple in SBT Patient only	Not specified	Before SBT 37 (± 32) End of SBT 60 (± 35)	Pharmacological: Inhaled beta-2-stimulants and steroids Suction to remove airway secretions Oxygen delivery devices: Oxygen therapy delivery Ventilator management: PSV (PS 21 ± 3 cmH_2_0 & PEEP < 6 cmH_2_0) Change of ventilator pressure settings and volumes

*AC* assist-control, *A-C-F* acute-on-chronic, *ACV* assist-control ventilation, *AE* acute exacerbation, *APRV* airway pressure release ventilation, *ARDS* acute respiratory distress syndrome, *ARF* acute respiratory failure, *BIPAP* biphasic positive airway pressure, *CO* carbon monoxide, *CF* cystic fibrosis, *CHF* congestive cardiac failure, *COPD* chronic obstructive pulmonary disease, *CPAP* continuous positive airway pressure, *CPO* cardiogenic pulmonary oedema, *CSV* continuous spontaneous ventilation, *DNMD* decompensation of neuromuscular disease, *HFNOT* high-flow nasal oxygen therapy, *HSCT* haematopoietic stem cell transplantation, *ICU* intensive care unit, *ICU’s* intensive care units, *ILD* interstitial lung disease, *IPAH* idiopathic pulmonary arterial hypertension, *NAVA* neurally adjusted ventilatory assist, *NIMV* non-invasive mechanical ventilation, *NIV* non-invasive ventilation, *PAV +* proportional assist ventilation, *PEEP* positive end expiratory pressure, *PS* pressure support, *PSV* pressure support ventilation, *PSV-B* pressure support ventilation- baseline, *PSV-P* pressure support ventilation- personalisation, *RF* respiratory failure, *SIMV* Synchronised Intermittent Mandatory Ventilation, *SBT* spontaneous breathing trial, *VC-CMV* volume control continuous mandatory ventilation, *VC-IMV* volume control intermittent ventilation

All quantitative studies described the breathlessness assessment tool used: visual analogue scale (6/16) [6, 26, 31, 37, 40, 42]; numerical rating scale (3/16) [32, 38, 39]; modified BORG scale (5/16) [30, 34–36, 41]; visual analogue scale and Intensive care-Respiratory Distress Observation Scale (1/16) [28]; visual analogue scale and mechanical ventilation-respiratory distress observation scale (1/16) [33] and baseline dyspnoea index (1/16) [29]. The remaining quantitative study did not use a patient self-reported breathlessness assessment tool, but a retrospective search and review of nursing documentation for subjective terms including “dyspnea”, “shortness of breath” “breathlessness” and “the patient describing feeling breathless” alongside objective measures including oxygen saturations, arterial blood gas analysis, level of consciousness and screening for delirium [27].

A validated patient self-reported evaluation tool which provided data on the level of intensity of the breathlessness was used in 17/19 studies [6, 26, 28–42]. The visual analogue scale, (VAS) a continuous line ranging from 0 cm (no breathlessness) up to 10 cm (worst imaginable respiratory discomfort) was the most frequently used breathlessness intensity assessment tool in 6/15 studies [6, 26, 31, 37, 40, 42]. The Numerical Rating Scale, (NRS) numbers ranging from 0 (no difficulty breathing) up to 10 (worst difficulty breathing ever) was used in 3/15 studies [32, 38, 39]. The modified BORG scale (mBORG), a semi-ratio scale with some verbal descriptors and numbers ranging from 0 (no exertion) up to 10 (maximal) was used in 5/15 studies [30, 34–36, 41]. Clinically important breathlessness using these self-reported tools is defined as a VAS or NRS score of ≥ 4 [20] or mBORG score of ≥ 3 or “moderate” [20].

Only eight studies specified when the breathlessness assessment was undertaken: pre-SBT (1/8) [6], start and finish of SBT (2/8) [26, 28] during SBT (1/8) [35], end of SBT (2/8) [39, 42], pre and post-intervention- not specified (1/8) [29], pre / post-initiation of / NIV (1/8) [34].

In 17 studies the frequency of the breathlessness assessment was described and varied from once daily up to extubation (1/17) [6], once only (6/17) [28, 30, 32, 36, 37, 40], once daily over four days (1/17) [29], once daily on days 1, 2 and 5 (1/17) [37], once daily over 7 days (1/17) [31], once daily up to extubation (1/17) [6], twice only (1/17) [26], three times during single episode of intervention (2/17) [34, 35], once- not specified (1/17) [38] and multiple- not specified (2/17) [37, 41].

In patients receiving IMV (n = 14) [6, 26–28, 30–37, 39–41] the highest reported median VAS, NRS and mBORG scores were 6.2cm [28], 5 [39] and 6 [30] respectively. The interquartile range for VAS, NRS and mBORG were 0 cm [26, 31] to 10 cm [26]; 2 [39] to 7 [39] and 2.3 [29] to 7 [36] respectively. In patients receiving either NIV or HFOT (n = 4) [29, 31, 34, 38], the highest reported median (IQR) VAS, NRS and mBORG scores were 3cm (0 to 6) [31], 8 (5–10) [38] and 4 (3 to 5) [34] respectively.

Four studies included breathlessness intensity ratings from: patient, caregiver and nurse (1/16) [32]; patient, nurse, physician and respiratory therapist (mBorg) (1/16) [36], patients and their relatives [38]; and patient, nurse and physician (NRS) (1/16) [39]. In all studies, the clinicians underestimated the intensity of the breathlessness.

### Perceptions of routine breathlessness assessment and management by ICU Nurses

Qualitative findings from the one mixed-methods study [43] are summarised in Table 5. Six themes were presented including importance, implementation and practicalities of breathlessness assessment, patient-report *versus* observed signs, patients’ ability to rate breathlessness and interventions in response to breathlessness assessment [43].

**Table 5:** The results of the mixed methods study

Themes identified						
Author Year Country	Theme 1: *“Importance of dyspnoea assessment”*	Theme 2: *“Implementation of dyspnoea assessment”*	Theme 3: *“Practicalities of assessment”*	Theme 4: *“Patients report versus observed signs to assess dyspnoea”*	Theme 5: *“Patient’s ability to rate dyspnoea”*	Theme 6: *“Interventions after dyspnoea assessment”*
Baker 2020 [42] USA	The nurses rated the following as important or very important: – 70% to use a uniform tool – 86% to track every shift – 73% in improving patient-centred care – 70% at predicting outcome	92% of the nurses found the NRS easy or very easy to administer 68% of the nurses reported the NRS had no impact on workflow 32% of the nurses reported improved workflow	76% of the nurses first asked their patients a yes / no question: *“Are you having breathing difficulty?”* In patients who responded no, 42% of the nurses recorded a score 0, without asking the patient to self-rate a score	42% of the ICU nurses reported using a combination of patient reports and observed signs of dyspnoea	70% of the ICU Nurses reported that patients gave meaningful rating for dyspnoea at least half of the time 81% of the ICU Nurses reported that patients gave meaningful rating for pain at least half of the time	95% of the ICU nurses reported that for patients reporting increasing shortness of breath, non-pharmacological interventions would be employed 60% of ICU nurses reported assessing for the potential need of narcotics
	Quotes: *“I have always completed the dyspnea assessment when I assess respiratory distress.”* *“Dyspnea assessment was already part of my patient assessment if the patient was able to report their level of respiratory distress.”* *“Allows for patient to explain in their own words how they are feeling.”*	Quotes: *“There are too many options for the different levels of distress... mild, moderate, and severe would suffice.”* *“Our patients often cannot rate/score their dyspnea. They don’t understand the scale.”* *“Make the scale simpler... normal, worse than normal, worse than it’s ever been before.”*	Quotes: *“I typically ask if they are having difficulty breathing or feeling short of breath. If the answer is no, I presume that the number rating is 0/10 as I would presume with the pain scale”* *“A patient with COPD* *may say no, but their* *baseline dyspnea score could be 4, so it is* *important to obtain the baseline report.”*	Quotes: *“Often patients are intubated, confused, delirious or have dementia and cannot answer”* *“In the ICU, most patients cannot speak due to the ventilator or altered mental status... it is important to use nonverbal cues from the patient to assess.”*	Quotes: No quotes provided by the researchers	Quotes: No quotes provided by the researchers

Most (70%) ICU nurses reported that using a uniform breathlessness assessment tool in the ICU was either important or very important, consistent with comments; “*I have always completed the dyspnoea assessment when I assess respiratory distress”* and *“Dyspnoea assessment was already part of my patient assessment if the patient was able to report their level of respiratory distress”* [43]*.* Likewise, 73% reported that using a breathlessness assessment tool helped to improve the delivery of patient centred care, commenting; *“Allows for patient to explain in their own words how they are feeling”* [43]*.*

Nearly all (92%) ICU nurses reported that the NRS was easy to use, and either did not impact (68%) or improved (32%) workflow. However, a significant minority had the following concerns about the implementation of breathlessness assessment *“There are too many options for the different levels of distress”, “Our patients often cannot rate their dyspnoea. They don’t understand the scale*” and *“Make the scale simpler... normal, worse than normal, worse than it’s ever been before”* [43].

Three quarters of the ICU nurses initially assessed their patients by asking a ‘yes / no’ question; *“are you having breathing difficulty?”* [43]*.* Nearly half of these nurses reported that if a patient responded ‘no’, recorded an NRS score of 0 without further inquiry; *“I typically ask if they are having difficulty breathing or feeling short of breath. If the answer is no, I presume that the number rating is 0/10 as I would presume with the pain scale”.* However, some ICU nurses recognised the importance of getting a baseline breathlessness score for their patients, especially when managing patients with chronic respiratory conditions; *“A patient with COPD [chronic obstructive pulmonary disease] may say no, but their baseline dyspnoea score could be 4, so it is important to obtain the baseline report”* [43]*.*

Almost half of the ICU nurses used a combination of patient-reported and observed signs to assess breathlessness because many did not have capacity to communicate clearly; *“often patients are intubated, confused, delirious or have dementia and cannot answer”* and *“In the ICU, most patients cannot speak due to the ventilator or altered mental status... it is important to use nonverbal cues from the patient to assess”* [43]. However, the survey data showed that most ICU nurses recognised that at least half of the time ICU patients could self-report and give a meaningful rating of their own breathlessness and pain respectively [43].

### Management of breathlessness in the ICU

Details of the breathlessness management approaches was provided by 6/19 studies [3, 28, 29, 32, 41, 42] only one of which included patients receiving NIV [29]. The pharmacological treatment approaches described included opioids (1/6) [32], bronchodilators (2/6) [32, 42] and benzodiazepines (1/6) [32]. The non-pharmacological treatment approaches included suction to remove airway secretions (1/6) [42], ventilator management (5/6) [28, 32, 33, 41, 42], ventilator / patient optimisation (1/6) [28], back massage (1/6) [29], patient repositioning (2/6) [29, 32], oxygen delivery device management including increasing / decreasing fraction of inspired oxygen and change of delivery device (2/6) [32, 35], repositioning and coaching the patient to take slow / deep breaths, and the pharmacological interventions [43].

## Discussion

This rapid review presents data from 2822 critically ill adult patients managed in the ICU receiving HFOT, NIV and IMV. We found that although mechanical ventilation aims to relieve breathlessness in patients, breathlessness in mechanically ventilated ICU patients is common, with prevalence varying depending the population, timing and assessment tools used. Intensity scores indicate moderate to severe breathlessness, but it is underestimated and undertreated by clinicians. This risks unalleviated suffering for survivors (both during their ICU stay, and in the longer-term following discharge), for those who are dying, and for family carers witnessing such suffering. Untreated breathlessness may also present a potential barrier for patients being offered rehabilitation although we found no data describing this.

### Prevalence of breathlessness in the ICU

We found prevalence estimates of breathlessness comparable to other equally distressing symptoms. The weighted mean prevalence of breathlessness in patients receiving IMV was 49%, and prior to the initiation mean prevalence of NIV was 55%.

The prevalence of moderate to severe pain at rest in the adult ICU population is approximately 50%, and higher (80%) during procedures commonly delivered in the adult ICU [45, 46]; comparable to prevalence of other key distressing symptoms. Agitation is reportedly present in 50% to 70% of all adults either on admission to ICU or developing several days later [47]. Up to one third of all adult ICU patients develop delirium [48], especially those receiving invasive ventilation [47]. Sleep disturbance is high, reported by 60% of ICU survivors [49]. Immobility in critically ill patients leads to rapid and early muscle wasting; 30% occurring within the first 10 days of admission [50] and up to half of all ICU survivors experiencing ICU-acquired weakness which has short and long-term adverse impacts [51, 52].

Systematic assessment and management of pain, agitation, delirium, sleep disturbance and immobility are included in current evidence-based clinical guidelines [7] implemented using the Assess, prevent and manage pain; Both spontaneous awakening and spontaneous breathing trials; Choice of sedation and analgesia; Delirium: assess, prevent and manage; Early mobility and exercise; Family engagement and empowerment, (ABCDEF) care bundle [53]. The ABCDEF care bundle is applicable to every adult ICU patient irrespective of their diagnosis and reason for admission, and short-term positive outcomes have been shown relating to survival, coma, delirium, mechanical ventilation usage, restraint-free care, ICU readmissions and post-ICU discharge location [54]. Breathlessness is notable by its absence in the ABCDEF care bundle or in any current critical care guidelines [7]. A baseline universal assessment of dyspnoea using the NRS on admission to hospital is feasible and can help identify patients at risk of future harm in the acute ward setting [55].

### Identification, assessment and management

The current critical care guidelines provide clear, evidence-based recommendations for the management of pain, agitation, sleep disturbance, delirium and immobility which have reported short-term positive outcomes relating to a range of measures [7, 56]. Our rapid review shows that, in contrast, breathlessness assessment in the ICU varies greatly in terms of the timing, frequency and choice of assessment tool used.

The recommended approach to assessing breathlessness in adults is to use a patient self-reported tool where possible, rather than relying on clinical signs of respiratory distress only [4]. The lack of adequate identification and assessment of breathlessness is a barrier to individualised holistic management of both patients with potential for rehabilitation and for those who are dying.

In the four studies that compared clinician and patient assessments, ICU clinicians consistently underestimated the presence an intensity of breathlessness [32, 36, 38, 39] and identification of breathlessness did not necessarily translate into attempts to alleviate it. This is consistent with under-management reported in the wider literature [57, 58] and is not exclusive to the ICU. An RCT demonstrated that respiratory clinicians were less likely to consider further management for persistent breathlessness compared to chronic pain in patients with COPD and optimised disease-related treatment [59]. A cohort study examining the prevalence and management of breathlessness in COPD patients found that despite persistent breathlessness being apparent in around half of admissions, there was little evidence of any breathlessness-targeted treatment [60]. Breathlessness has been described as “invisible” to clinicians and the wider healthcare system [61, 62]. In a large (n = 10,000) population-based study, 11% respondents described daily limiting breathlessness (mMRC ≥ 2), of whom about a third had not raised this symptom with their clinician. For 85% of these, their clinician had not asked about breathlessness either [61]. UK-based specialist respiratory trainees describe it difficult to talk about breathlessness with their patients due to perceived therapeutic nihilism, and a lack of awareness of other services and time pressures [58], mirroring findings from other world settings [63].

### Clinical importance

Adults receiving HFOT, NIV and IMV in the ICU, self-report breathlessness that is clinically significant for those with potential for recovery and those who are dying. Despite overall improving survival rates, a growing population of patients discharged from the ICU develop post-intensive care syndrome, including cognitive, mental and physical health problems [64, 65]. Breathlessness could play a contributory role in this situation by delaying effective and timely rehabilitation in the ICU, potentially exacerbating functional impairment [15, 18, 66, 67]. During ICU admission, breathlessness causes immediate distress and feelings of anxiety, helplessness, fear and existential threat [6, 68, 69], compounded by barriers to verbal communication [70]. In the longer-term, repeated suffering could cause post-traumatic stress disorder, (PTSD) [6]. PTSD affects approximately 20% of ICU survivors [65, 71], with implications for family caregivers. Observing a loved one experiencing breathlessness is distressing—whether they have potential for recovery or are dying—and may even induce vicarious breathlessness in the caregiver [72].

### Strengths and limitations

We conducted our rapid review using methods recommended by the Cochrane Rapid Reviews Methods Group Recommendations [21] and reported in conjunction with the Preferred Reporting Items for Systematic Reviews and Meta-Analyses 2020, (PRISMA) statement [22]. The demographic characteristics of the patient-participants, reason for critical care admission and the respiratory support provided are applicable to the wider critical care population commonly encountered in everyday clinical practice in the UK [73] and elsewhere.

We draw conclusions about treatment but recognise that in most studies the aim was not to relieve breathlessness, but only to assess it. Efforts to treat may have occurred but not documented as a study outcome.

Although rapid reviews may produce similar results and conclusions [74], systematic reviews remain the gold standard for providing evidence synthesis. They do not have the inherent limitations relating to the rapid review methodology [75]. In addition, we were unable to conduct a meta-analysis due to study heterogeneity. This brings potential inconsistency and difficulty in comparing results, limiting the robustness of the review’s conclusions. We may have missed relevant studies. In particular, the date limit of 2013, chosen to reflect current clinical practice in adult critical care, means we may have missed relevant earlier. Most primary studies were observational studies with methodological limitations and should be interpreted with caution. In addition, COVID-19 related articles were excluded as they represent a distinct population requiring particular management in the ICU, and largely relate to a specific duration in time. A brief scope of the literature indicated that most COVID-19 articles in the ICU setting did not focus on identification and assessment of breathlessness, or specifically its management during the patients’ time in the ICU, however, we may have missed relevant articles. There was a literature about management of breathlessness post-ICU, but this was out of scope.

Lastly, our review was unable to extensively address potential barriers or challenges to implementation of initiatives to systematically identify, assess and manage breathlessness in the ICU setting.

### Implications for clinical practice and policy

Adults needing ICU experience difficulty in communication, discomfort, pain, agitation, delirium, fear, anxiety, thirst, immobility and breathlessness [10]. The inclusion of the assessment and management for most of these symptoms in critical care evidence-based guidelines has revolutionised how adult patients are managed in this setting along with improved outcomes for the included symptoms [53, 54]. As breathlessness is as prevalent, clinically significant, but consistently underestimated and undermanaged by clinicians in the ICU the current critical care guidelines should be updated to include breathlessness [7].

Failure to include patient-report measures for intensity as well as presence risks underestimating both. All clinicians, in and beyond the ICU, should be able to use an appropriate self-reported rating tool which accommodates the patient’s own communication abilities. Demoule et al. [20] propose a breathlessness assessment algorithm taking this into account, using open-ended screening questions followed by a self-reported breathlessness tool for communicative patients, and the RDOS for those who cannot [20].

Two key recommendations suggested by Guttormson and colleagues are relevant: (i) assume that ICU patients are likely to experience all common symptoms, including breathlessness, and, (ii) make patient-centred plans to dynamically assess and manage these [10]. Breathlessness management plans also need to incorporate both pharmacological and non-pharmacological interventions [8].

Successful implementation would need a change in culture and provision of additional education and training for all ICU multi-disciplinary team members [10].

### Implications for future research

We highlight gaps in the literature. Firstly, we need a clearer understanding of the barriers and facilitators to implementing systems to ensure breathlessness is recognised and managed by all clinicians in the ICU setting [10]. Secondly, testing of the breathlessness assessment and management models presented by Demoule and colleagues to establish safety, efficacy and acceptability in patients receiving HFOT, NIV and IMV in the ICU is needed [10, 20]. Thirdly, trials evaluating the benefit of complex non-pharmacological breathlessness interventions known to be effective in other health-care settings should be conducted in the ICU, for patients with potential for recovery and for those who are dying. These should incorporate valid measures examining health related quality of life, functional status, symptom control and psychological distress. Fourthly, we found no literature exploring the impact of breathlessness on ICU rehabilitation. Given the likely relationship between the two, studies should formally investigate this issue. Finally, using quality improvement and implementation approaches the sustainability of introducing breathlessness assessment and management models are needed to establish and determine whether these can be delivered safely and consistently in the everyday clinical critical care setting [10, 20].

## Conclusion

Breathlessness in adults receiving non-invasive and invasive ventilation in the ICU is prevalent, clinically important, consistently underestimated and undermanaged by ICU clinicians. This disadvantages both those who will recover and those who will not. Whereas other symptoms are included in care bundles designed to identify, assess and manage distress, breathlessness is conspicuous by its absence. Our findings challenge practice in ICUs around the world and an urgent review of current critical care guidelines is needed.

### Supplementary Information

Additional file 1.

## Data Availability

All data are previously published in the included papers.
