# Supplementary material for: Breathlessness assessment, management and impact in the intensive care unit: a rapid review and narrative synthesis
Source: Ann Intensive Care. 2024 Jul 5;14:107. doi: 10.1186/s13613-024-01338-7 (PMC11229436; doi:10.1186/s13613-024-01338-7)
Supplement: Supplementary file 1 — Additional file 1. [file 13613_2024_1338_MOESM1_ESM.docx]

**Online Supplement Table 1:** Cohort Studies

|  | **Quality Appraisal Questions [16]** | | | | | | | | | | | |
| --- | --- | --- | --- | --- | --- | --- | --- | --- | --- | --- | --- | --- |
|  | **Question 1** | **Question 2** | **Question 3** | **Question 4** | **Question 5** | **Question 6** | **Question 7** | **Question 8** | **Question 9** | **Question 10** | **Question 11** | **Question 12** |
| **Author**  **Year**  **Country** | **Did the study address a clearly focussed issue?** | **Was the cohort recruited in an acceptable way?** | **Was exposure accurately measured to minimise bias?** | **Was the outcome accurately measured to minimise bias?** | **Have the authors identified all important cofounding factors?** | **Have they taken account of the cofounding factors in the design and/or analysis?** | **Was the follow up of subjects complete enough?** | **Was the follow up of subjects long enough?** | **Were appropriate statistical analysis methods used?** | **Do you believe the results?** | **Can the results be applied to the local population?** | **Do the results of this study fit with other available evidence?** |
| **Demoule**  **2022**[19]  France | Yes | Yes | Yes | Yes | Yes | Yes | Yes | Yes | Yes | Yes | Yes | Yes |
| **Atrous**  **2020**[23]  Egypt | Unclear – no timeframe | Yes | Yes | No, only in response to observed change | Yes | Yes | Unclear – not stated | Unclear – not stated | Yes | Unclear | Unclear | Yes |
| **Mazeraud**  **2020**[24]  Egypt | Yes | Yes | Yes | Yes | Yes | Yes | Yes | Yes | Yes | Yes | Yes | Yes |
| **Raux**  **2019**[26]  France | Yes | Yes- for an exploratory study | Yes | Yes | No, exploratory | No, exploratory | Yes | Yes | Yes  exploratory | Yes | Unclear, exploratory | Yes |
| **Dangers**  **2018**[27]  France  Belgium | Yes | Yes | Yes | Yes | Yes | Yes | Yes | Yes | Yes | Yes | Yes | Yes |
| **Haugdahl**  **2015**[31]  Norway | Yes | Unclear: not screened at weekends | Yes | Yes | Yes | No | Yes | Yes | No adjustment for confounders | unclear | Yes | Yes |

**Online Supplement Table 2:** Cross Sectional Studies

|  | **Quality Appraisal Questions [17]** | | | | | | | |
| --- | --- | --- | --- | --- | --- | --- | --- | --- |
|  | **Question 1** | **Question 2** | **Question 3** | **Question 4** | **Question 5** | **Question 6** | **Question 7** | **Question 8** |
| **Author**  **Year**  **Country** | **Were the criteria for inclusion in the sample clearly defined?** | **Were the study subjects and the setting described in detail?** | **Was the exposure measured in a valid and reliable way?** | **Were objective, standard criteria used for measurement of the condition?** | **Were confounding factors identified?** | **Were strategies to deal with confounding factors stated?** | **Were the outcomes measured in a valid and reliable way?** | **Was appropriate statistical analysis used?** |
| **Bureau**  **2022**[XX]  France | Yes | Yes | Yes | Yes | Yes | Yes | Yes | Yes |
| **Sato**  **2022**[20]  Japan | Yes | Yes | Yes | Yes | Yes | Yes | No | Yes |
| **Bureau**  **2021**[21]  France | Yes | Yes | Yes | Yes | Yes | Yes | Yes | Yes |
| **Gentzler**  **2019**[XX]  USA | Yes | Yes | Yes | Yes | Yes | Yes | Yes | Yes |
| **Binks**  **2017**[28]  USA | Yes | Yes | Yes | Yes | Yes | No, but exploratory small sample | Yes | Yes |
| **Persichini**  **2015**[XX]  France | Yes (note data collection only weekdays) | Yes | Yes | Yes | Yes | Yes | Yes (note data collection only weekdays) | Yes |
| **Baker**  **2020**[34]  USA  (survey data) | Yes | No | Yes | No | No | No | Yes | Yes |

**Online Supplement Table 3:** Single-Centre Cross-over Randomised Controlled Trials

| **Quality Appraisal Questions [16]:** | | **Akoumianaki**  **2017**[28]  Switzerland | **Fortis**  **2015**[XX]  USA | **Vitacca**  **2014**[33]  Italy |
| --- | --- | --- | --- | --- |
| **Question 1** | **Did the study address a clearly focused research question?** | Yes | Yes | Yes |
| **Question 2** | **Was the assignment of participants to interventions randomised?** | Yes | Yes | Yes |
| **Question 3** | **Were all participants who entered the study accounted for at its conclusion?** | Yes | Yes | Yes |
| **Question 4** | **Were the participants ‘blind’ to intervention they were given?** | Yes | Yes | Yes |
| **Question 5** | **Were the investigators ‘blind’ to the intervention they were giving to participants?** | Yes | Unclear | Unclear |
| **Question 6** | **Were the people assessing / analysing outcome/s ‘blinded’?** | No | Unclear | Unclear |
| **Question 7** | **Were the study groups similar at the start of the randomised controlled trial?** | No | No | Unclear |
| **Question 8** | **Apart from the experimental intervention, did each study group receive the same level of care (that is, were they treated equally)?** | Unclear | Yes | Yes |
| **Question 9** | **Were the effects of intervention reported comprehensively?** | Yes | No | Yes |
| **Question 10** | **Was the precision of the estimate of the intervention or treatment effect reported?** | No | No | No |
| **Question 11** | **Do the benefits of the experimental intervention outweigh the harms and costs?** | Unclear | Unclear | Unclear |
| **Question 12** | **Can the results be applied to your local population/in your context?** | Unclear | Unclear | Unclear |
| **Question 13** | **Would the experimental intervention provide greater value to the people in your care than any of the existing interventions?** | Unclear | Unclear | Unclear |

**Online Supplement Table 4:** Randomised Controlled Clinical Trials

| **Quality Appraisal Questions [16]:** | | **Yilmaz**  **2021**[22]  Turkey | **Demoule**  **2016**[30]  France |
| --- | --- | --- | --- |
| **Question 1** | **Did the study address a clearly focused research question?** | Yes | Yes |
| **Question 2** | **Was the assignment of participants to interventions randomised?** | Yes | Yes |
| **Question 3** | **Were all participants who entered the study accounted for at its conclusion?** | Yes | Yes |
| **Question 4** | **Were the participants ‘blind’ to intervention they were given?** | No | Yes |
| **Question 5** | **Were the investigators ‘blind’ to the intervention they were giving to participants?** | No | No |
| **Question 6** | **Were the people assessing / analysing outcome/s ‘blinded’?** | No | Yes |
| **Question 7** | **Were the study groups similar at the start of the randomised controlled trial?** | Yes | Yes |
| **Question 8** | **Apart from the experimental intervention, did each study group receive the same level of care (that is, were they treated equally)?** | Unclear | Yes |
| **Question 9** | **Were the effects of intervention reported comprehensively?** | Yes | Yes |
| **Question 10** | **Was the precision of the estimate of the intervention or treatment effect reported?** | No | Yes |
| **Question 11** | **Do the benefits of the experimental intervention outweigh the harms and costs?** | Unclear | Yes |
| **Question 12** | **Can the results be applied to your local population/in your context?** | Unclear | Yes |
| **Question 13** | **Would the experimental intervention provide greater value to the people in your care than any of the existing interventions?** | Unclear | Yes |

**Online Supplement Table 5** Qualitative data in Baker et al 2020

|  | **Quality Appraisal Questions [16]** | | | | | | | | | |
| --- | --- | --- | --- | --- | --- | --- | --- | --- | --- | --- |
|  | **Question 1** | **Question 2** | **Question 3** | **Question 4** | **Question 5** | **Question 6** | **Question 7** | **Question 8** | **Question 9** | **Question 10** |
| **Author**  **Year**  **Country** | **Are the results valid?** | **Is a qualitative methodology appropriate?** | **Was the research design appropriate to address the aims of the research?** | **Was the recruitment strategy appropriate to the aims of the research?** | **Was the data collected in a way that addressed the research issue?** | **Has the relationship between researcher and participants been adequately considered?** | **Have ethical issues been taken into consideration?** | **Was data analysis sufficiently rigorous?** | **Is there a clear statement of findings?** | **Will the results help locally?** |
| **Baker**  **2020**[34]  USA | Yes | Yes | Yes | Unclear | Yes | Unclear | Unclear | No | Yes | Unclear |
